# Clinical Course of a Child With Cystic Fibrosis and the Genotype F508del/CFTRdup1_11: A Case Report

**DOI:** 10.7759/cureus.67792

**Published:** 2024-08-26

**Authors:** Argyri Petrocheilou, Maria Tzetis, Ioanna Loukou

**Affiliations:** 1 Cystic Fibrosis Department, Agia Sofia Children's Hospital, Athens, GRC; 2 Medical Genetics Department, University of Athens Medical School, Athens, GRC

**Keywords:** f508del, cftr, cftrdup1_11, newborn screening, cystic fibrosis

## Abstract

Cystic fibrosis (CF) is a hereditary disease with great genetic complexity as not all mutations are disease-causing and genotype doesn’t always predict phenotype. This case involves a child with CF and genotype F508del/CFTRdup1_11. The CFTRdup1_11 duplication was not reported previously, and genetic counseling was based on reports describing the clinical course of people carrying smaller duplications of the same area combined with F508del. The predicted clinical presentation was CF with pancreatic insufficiency. However, the case presented has so far shown no clinical symptoms and has borderline sweat chloride concentrations.

## Introduction

Cystic fibrosis (CF) is a common genetic disease (autosomal recessive) that affects mostly people of Caucasian descent [1]. The genetics of the disease are complex, as more than 2000 mutations have been identified so far but not all of them are disease-causing [1]. Moreover, variants can cause more than one molecular defect, and modifier genes also play an important role [2]. Prenatal screening is often offered for this disease; however, genetic counseling is often challenging as the correlation between genotype and phenotype is not always predictable. This case involves a child with CF who exhibits a milder clinical presentation than predicted based on genotype.

## Case presentation

**Figure 1:**
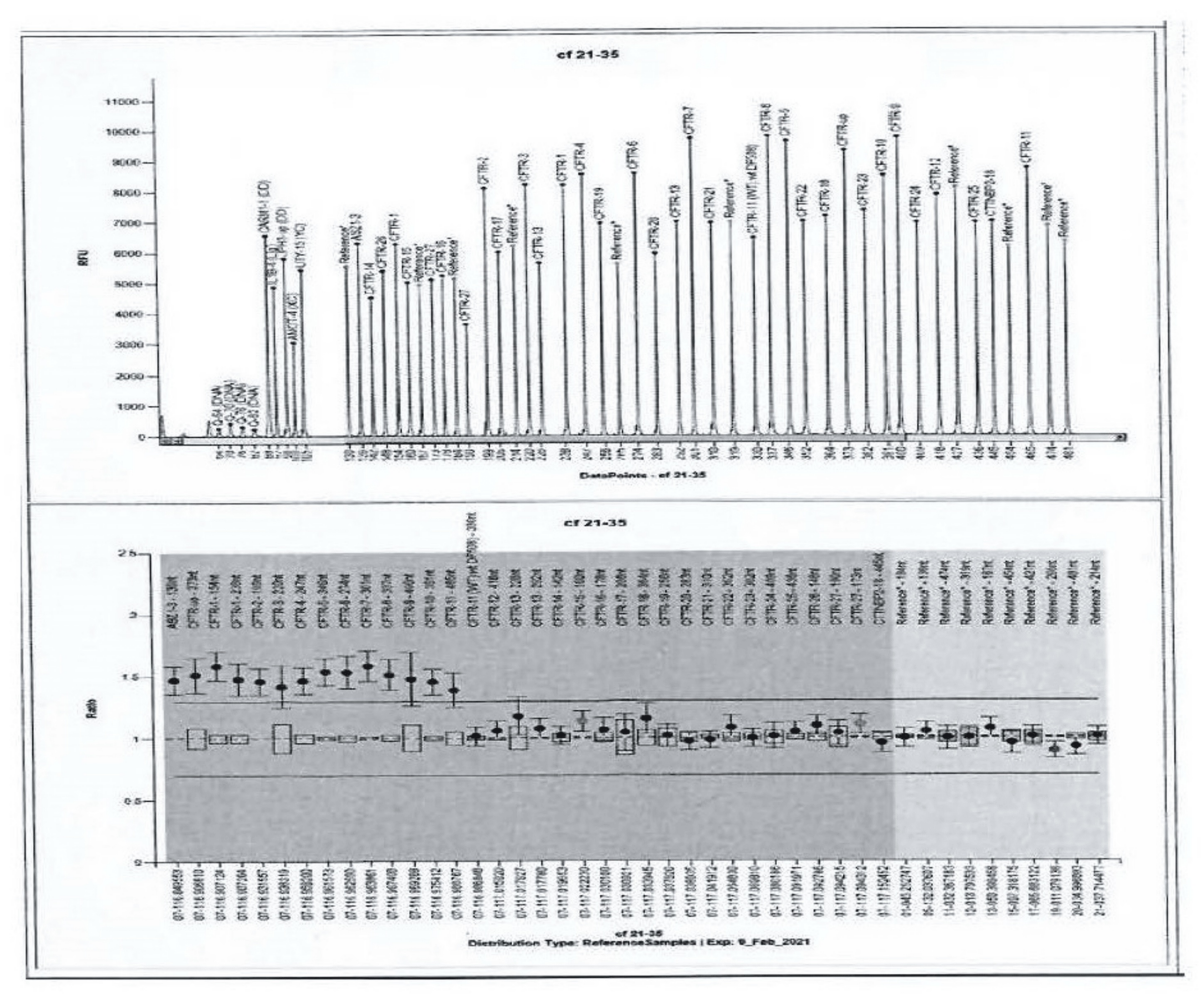
MLPA result MLPA: multiplex ligation-dependent probe amplification

This case presents a three-year-old boy with two CF transmembrane conductance regulator (CFTR) mutations (F508del/CFTRdup1_11) identified prenatally. The mother’s routine prenatal screening revealed that she was a carrier for F508del and that prompted paternal CFTR genetic testing. The father was identified as a carrier of CFTRdup1_11; therefore, genetic testing of the fetus (amniotic fluid) was advised. The fetus was found to be a composite heterozygote for F508del/CFTRdup1_11 and the parents were referred for genetic counseling. This combination is not reported in CFTR2 or other clinical databases. However, as smaller duplications in the same exon, when combined with F508del, are associated with CF phenotype; the parents were informed that this combination would probably also be a CF disease-causing genotype. After discussion with the genetic and CF care teams, the parents decided to continue the pregnancy, and the infant was born at term. The genotype was confirmed by multiplex ligation-dependent probe amplification (MLPA) (P091-CFTR) at the University of Athens Medical School Genetics Laboratory postnatally, with the gene map shown in Figure 1. The infant's weight was 3980 grams, and the perinatal period was uneventful. Neonatal screening was offered, and the immunoreactive trypsinogen (IRT) test was negative (53 mcg/L with a cutoff of 110 mcg/L), with the genotype confirmed. The infant was seen in the CF clinic at two weeks of age and was doing well. There were no signs of steatorrhea, the clinical examination was unremarkable, the sweat test results were borderline (chloride (Cl) = 33.8 mmol/L), and stool elastase levels were normal (1047 mcg/g).

**Figure 2:**
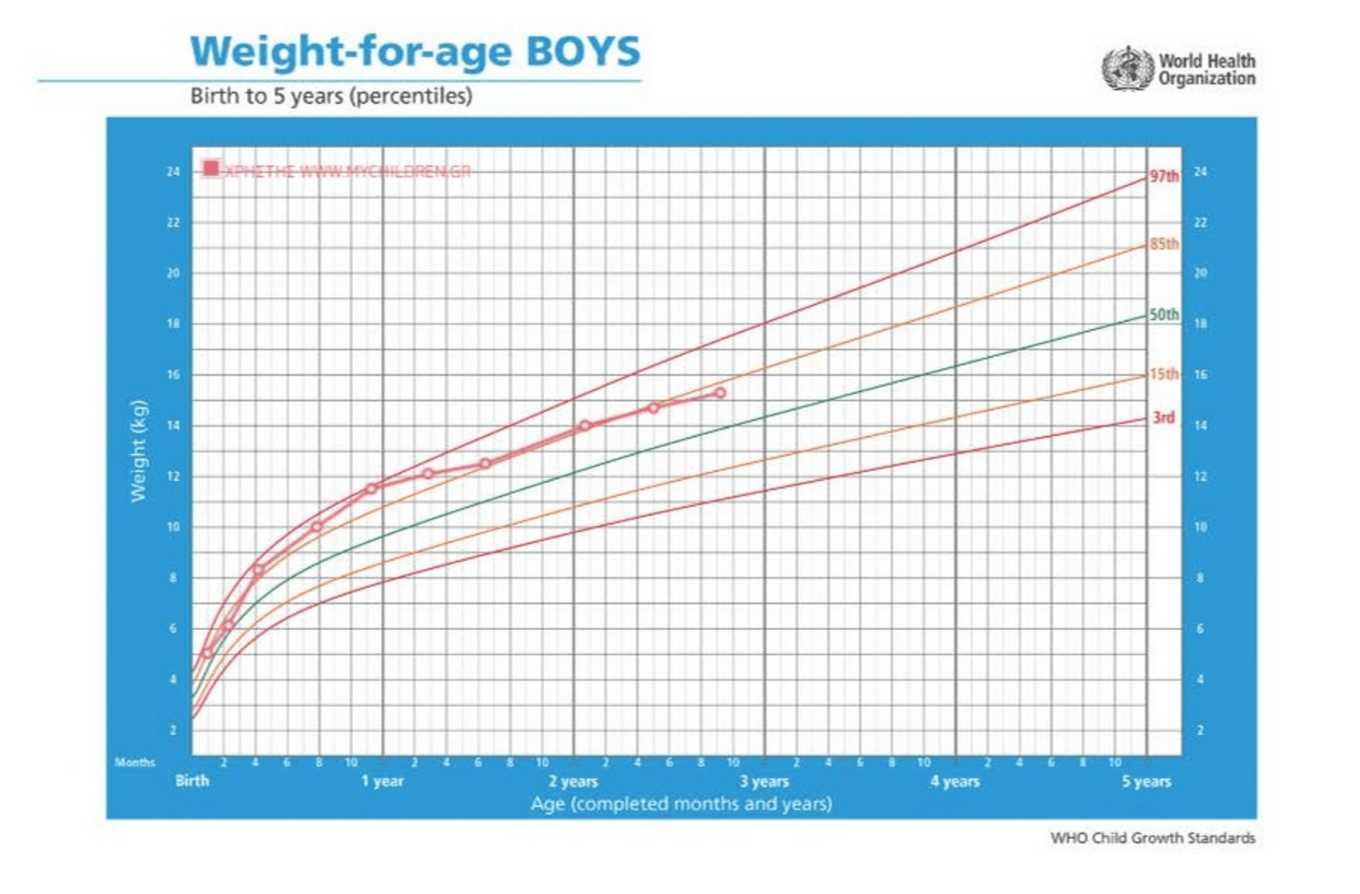
The patient's weight growth curve

**Figure 3:**
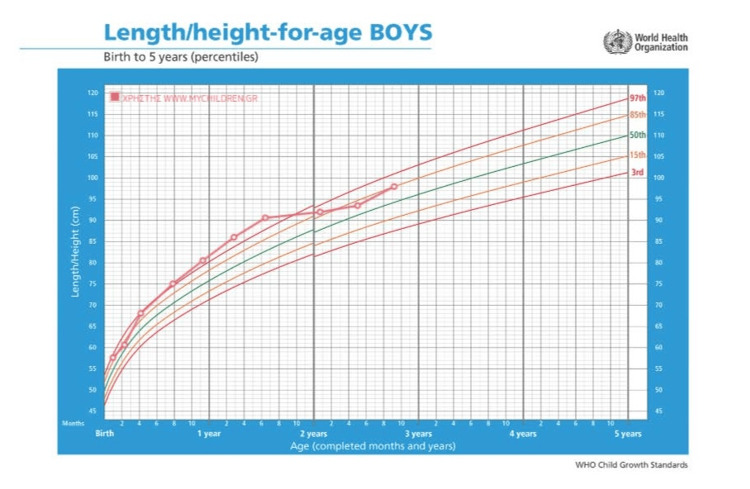
The patient's height growth curve

The infant is regularly followed in the CF clinic and is growing and developing normally so far. Stool elastase was not repeated as the child has been growing well and has no malabsorption symptoms. Respiratory cultures are being performed approximately every three months and usually grow normal flora. Positive cultures for *Staphylococcus aureus* were successfully treated with amoxicillin/clavulanate per os and *S. aureus *has not been isolated in the last 18 months. Two cultures were positive for *Morganella morganii*, one for *Enterobacter cloacae*, and one for *Candida parapsilosis*. These microbes were not considered pathogens and were not treated with antibiotics. No respiratory symptoms were reported with any of the cultures. There have been no admissions to the hospital thus far, and his growth has been tracking along the 80th-85th percentile (Figures 2-3).

The sweat test was repeated at three years of age, and the result remained borderline (sweat chloride = 31 mmol/L) (Table 1). Parents of the child involved had provided their informed consent prior to inclusion in this case presentation.

**Table 1:** Patient initial laboratory tests

Laboratory tests	Value	Normal values	Interpretation
Immunoreactive trypsinogen (IRT)	53	<110 mcg/L	Negative
Stool elastase	1047	>200 mcg/g	Pancreatic sufficiency
Initial sweat chloride	33	Negative <30 mmol/L; Borderline 30-60 mmol/L; Positive >60 mmol/L	Borderline
Repeat sweat chloride	31	Negative <30 mmol/L; Borderline 30-60 mmol/L; Positive >60 mmol/L	Borderline

## Discussion

To our knowledge, this is the first report of the F508del/CFTRdup1_11 composite heterozygote. Smaller duplications, such as CFTRdup 6b_10 in combination with F508del, have been associated with a CF phenotype, characterized as CF-causing as expected to result in little or no CFTR protein. Therefore, the patient's genotype was assumed to also be CF-causing. The child has been growing well and has no signs or symptoms of CF so far. This child has one CF-causing mutation (F508del) and a duplication not reported in clinical databases that were presumed to be CF-causing based on similar duplications, a reportedly negative newborn CF screen, two borderline sweat chloride measurements and so far no clinical symptoms or signs of CF. According to the Cystic Fibrosis Foundation (CFF) diagnosis consensus guidelines [3] and to the recently published Chinese experts' consensus statement for diagnosis and treatment of CF [4], the diagnosis of classic CF can't be made in this child. Further follow-up will determine whether this is a case of CF or cystic fibrosis screen positive inconclusive diagnosis (CFSPID). It is also possible that symptoms could develop later in life, as seen in cases of monosymptomatic CF.

Prenatal screening followed by genetic counseling for CF is common in many countries, but genetic counseling for CF can be challenging because the phenotype doesn't always correlate with the genotype. It is well-documented that in silico models can only be used as an indication to assist with phenotype prediction, and novel variants continue to be identified [5]. Databases such as CFTR2 and guidelines [6,7] help with phenotype prediction, however, especially in the case of rare variants, the addition of more clinical data and follow-up data leads to the reclassification of variants. The role of modifier genes also has to be elucidated on differences in phenotype. Newborn screening has also increased the number of variants of unknown significance as infants are identified prior to developing symptoms [8]. Follow-up data on children with CFSPID have demonstrated an 11%-38% reclassification rate to CF [3]. The challenge of genetic counseling is increasing in the modern era of new therapeutic options, as genetic counselors are often asked to predict the possibility of future therapies for each individual variant [8].

## Conclusions

It is often stated that each CF case is unique as the same mutations of the CFTR gene can result in different clinical presentations. Genetic counselors are often asked to predict the severity of clinical presentation prior to birth and in silico models are often used to predict clinical response to new therapies that are genotype-dependent. The case presented has no clinical symptoms so far and doesn't meet the criteria for a CF diagnosis, despite carrying a mutation combination that was predicted to cause CF with pancreatic insufficiency based on smaller duplications. This case adds to the pool of data indicating limitations to in silico models, as clinical phenotype can greatly differ from predicted. Caution is needed when interpreting prenatal genetic results.

## References

[1] Ong T, Ramsey BW (2023). Cystic fibrosis: a review. JAMA.

[2] Grasemann H, Ratjen F (2023). Cystic fibrosis. N Engl J Med.

[3] Farrell PM, White TB, Ren CL (2017). Diagnosis of cystic fibrosis: consensus guidelines from the Cystic Fibrosis Foundation. J Pediatr.

[4] (2023). Chinese experts consensus statement: diagnosis and treatment of cystic fibrosis (2023) [Article in Chinese]. Zhonghua Jie He He Hu Xi Za Zhi.

[5] Poulou M, Fylaktou I, Fotoulaki M, Kanavakis E, Tzetis M (2012). Cystic fibrosis genetic counseling difficulties due to the identification of novel mutations in the CFTR gene. J Cyst Fibros.

[6] Sosnay PR, Siklosi KR, Van Goor F (2013). Defining the disease liability of variants in the cystic fibrosis transmembrane conductance regulator gene. Nat Genet.

[7] Moskowitz SM, Chmiel JF, Sternen DL, Cheng E, Gibson RL, Marshall SG, Cutting GR (2008). Clinical practice and genetic counseling for cystic fibrosis and CFTR-related disorders. Genet Med.

[8] Foil KE, Powers A, Raraigh KS, Wallis K, Southern KW, Salinas D (2019). The increasing challenge of genetic counseling for cystic fibrosis. J Cyst Fibros.

